# Qualitative and quantitative analysis of lipid droplets in mature 3T3-L1 adipocytes using oil red O

**DOI:** 10.1016/j.xpro.2024.102977

**Published:** 2024-06-12

**Authors:** Isabell Kaczmarek, Tomáš Suchý, Martina Strnadová, Doreen Thor

**Affiliations:** 1 Rudolf Schönheimer Institute of Biochemistry, Medical Faculty, Leipzig University, Leipzig, 04103 Saxony, Germany

**Keywords:** Cell culture, Metabolism, Microscopy

## Abstract

By differentiating into mature adipocytes, 3T3-L1 cells can be utilized as a model cell line to investigate (pre)adipocyte function *in vitro*. Here, we present a protocol for combining qualitative and quantitative analysis of lipid droplets in mature 3T3-L1 adipocytes using oil red O. We describe steps to differentiate 3T3-L1 preadipocytes to adipocytes and give detailed procedures to determine total lipid amount as well as lipid droplet size and number using microscopic devices and an ImageJ macro.

For complete details on the use and execution of this protocol, please refer to Kaczmarek et al.^1^

## Highlights

• Differentiation of 3T3-L1 preadipocytes to adipocytes
• Assessing treatment-dependent changes on adipogenesis using oil red O staining
• Combination of quantitative and qualitative analysis of lipid accumulation

## Abstract (editor highlights)

Publisher’s note: Undertaking any experimental protocol requires adherence to local institutional guidelines for laboratory safety and ethics.

## Graphical abstract

**Figure:**
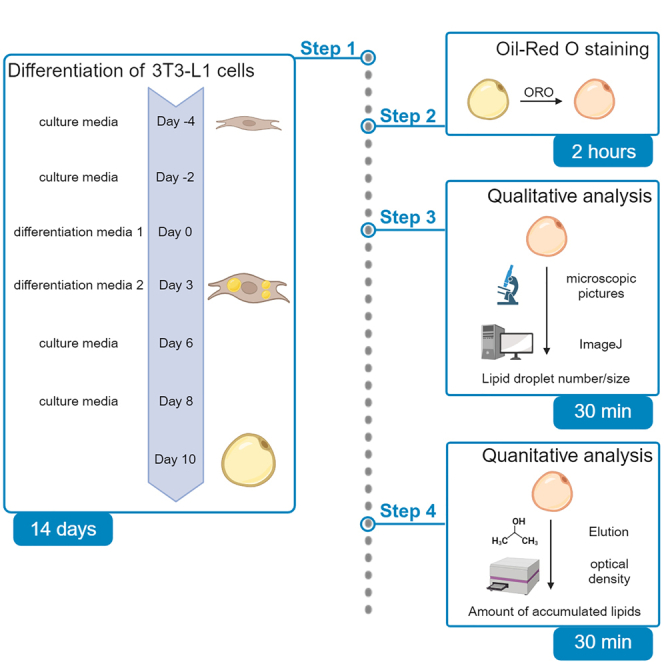

## Before you begin

3T3-L1 cells are a fibroblast cell line with adipogenic potential used as a model to study adipogenesis and adipocyte function *in vitro.*^2^ Incubation with a mixture of 3-Isobutyl-1-methylxanthine (IBMX), dexamethasone, and insulin leads to accumulation and storage of lipid droplets.^3^ IBMX increases intracellular cAMP levels by inhibiting phosphodiesterases and, thereby, increases PKA activity.^4^ Dexamethasone is necessary in the early phase of adipogenesis activating transcription factors of the C/EBP family.^5^ Insulin has a positive effect onto adipogenesis by stimulating glucose uptake and fatty acid synthesis as well as inducing transcription factors such as SREBP-1c and PPARγ.^6^ Furthermore, rosiglitazone, an agonist of the master regulator of adipogenesis – PPARγ – can be added during the differentiation process. Although in general rosiglitazone is dispensable for the differentiation process, several publications as well as our own experience demonstrate that differentiation is more pronounced and reliable when adding rosiglitazone.^7^

To assess adipogenesis, we use Oil Red O (ORO) staining of lipids in differentiated 3T3-L1 cells, which can be eluted and quantified by measuring the optical density (OD). However, reduction in lipid accumulation can have different causes such as a smaller number of droplets or smaller size of droplets. Therefore, qualitative analysis of ORO-stained lipid droplets^8^ will give further insights into the mechanisms leading to reduced differentiation. In the here presented protocol, we combine quantitative and qualitative analysis of 3T3-L1 differentiation using the same cells for both assays.

### Cell culture of 3T3-L1 cells

**Timing:** ∼**1 h**

The protocol is customized for 3T3-L1 cells seeded in 175 cm^2^ flask.^9^ If not stated otherwise, reagents can be substituted with alternatives from different vendors. All of the following procedures need to be performed under sterile conditions using a safety cabinet. Media and buffers used for cell culture should be prewarmed at 37°C.

***Note:*** Repeat this protocol every 48–72 h to avoid confluence higher than 80% of the cells.

1. Aspirate the old media.
2. Wash cells carefully.
  a. Add 5 mL of PBS.
    ***Note:*** Use PBS without Ca^2+^ and Mg^2+^.
  b. Gently shake the flask.
  c. Remove and discard PBS.
3. Detach the cells.
  a. Add 2 mL of 0.25% Trypsin/EDTA.
  b. Incubate for 1–2 min (37°C, 5% CO_2_).
  c. Tap the bottle with the flat of your hand to loosen the cells even better.
  d. Add 8 mL of culture media (CM) to stop trypsination.
4. Cell counting.
  a. Centrifugate cellular suspension for 5 min at 200 RCF.
  b. Discard the supernatant.
  c. Resuspend cells in 2 mL of CM.
  d. Count the cells using a Neubauer Glass Chamber (cell counting chamber).
5. Dilute cells in 20 mL CM/flask.
  a. Cultivation for 48 h: 2.50 × 10^5^ cells/flask.
  b. Cultivation for 72 h: 1.25 × 10^5^ cells/flask.

***Note:*** Evenly distribute cells by moving the flask.

6. Incubate cells for 2–3 days (37°C, 5% CO_2_).

### Download ImageJ software

**Timing:** ∼**10 min**

7. Download ImageJ using the following link: https://fiji.sc/.
8. Install ImageJ.

## Key resources table

**Table:** 

REAGENT or RESOURCE	SOURCE	IDENTIFIER
**Chemicals, peptides, and recombinant proteins**		

1x D-PBS	Thermo Fisher Scientific	14190144
Dexamethasone	Sigma-Aldrich	D4902
Dulbecco’s modified Eagle’s medium (DMEM)	Thermo Fisher Scientific	41966029
Fetal bovine serum (FBS)	Thermo Fisher Scientific	10270106
Formaldehyde solution	Carl Roth	4979.2
Insulin from bovine pancreas	Sigma-Aldrich	I6634
IBMX	Sigma-Aldrich	I5879
Isopropanol	Carl Roth	9265.2
Oil red O	Sigma-Aldrich	O-0625
Penicillin-Streptomycin (10,000 U/mL) (Pen/Strep)	Thermo Fisher Scientific	15140122
Rosiglitazone	Sigma-Aldrich	R2408
Trypsin-EDTA (0.25%), phenol red	Thermo Fisher Scientific	25200056

**Experimental models: Cell lines**		

3T3-L1 cells	ATCC	CL-173; RRID:CVCL_0123

**Oligonucleotides**		

Non coding (siNC), rCrGrUrUrArArUrCrGrCrGrUr ArUrArArUrArCrGrCrGrUAT	OriGene	SR30004
siFzd5, rGrCrArCrUrArArGrArCrGrGrArCrArArGr CrUrArGrArGAA	OriGene	SR417789, siRNA C

**Software and algorithms**		

BioRender	BioRender	https://biorender.com/
GraphPad Prism	GraphPad Software	https://www.graphpad.com/scientific-software/prism/
ImageJ 1.53q	Fiji	https://fiji.sc/

**Other**		

175 cm^2^ cell culture flask	Greiner Bio-One	658175
6-well plate	Greiner Bio-One	657160
12-well plate	Greiner Bio-One	665180
24-well plate	TPP Techno Plastic Products AG	92024
48-well plate	Greiner Bio-One	655180
96-well plate	Greiner Bio-One	677165
50 mL tubes	SARSTEDT GmbH	62547254
Filtropur S 0.2	SARSTEDT GmbH	83.1826.001
CO_2_ incubator for cell culture	Thermo Fisher Scientific	Heraeus Kendro HeraCell
Centrifuge	Thermo Fisher Scientific	Megafuge 16R
Improved Neubauer counting chamber	Th. Geyer GmbH	6261165
Compact fluorescence microscope BZ-X800	Keyence	Keyence bz-x800_long
Multimode multilabel plate reader to measure absorbance at 500 nm and 620 nm	PerkinElmer	EnVision 2104 multilabel plate reader with monochromator
Safety cabinets	Thermo Fisher Scientific	HERAsafe KS 12

## Materials and equipment

**Table:** Culture media (CM)

Reagent	Final concentration	Volume
DMEM	89% (v/v)	44.5 mL
FBS	10% (v/v)	5.0 mL
Penicillin/Streptomycin (Pen/Strep)	1% (v/v)	0.5 mL
**Total**	**N/A**	**50.0 mL**

Storage conditions: 4 °C., maximum storage time: 3 months.

**Note:** Heat inactivation of FBS is not necessary, but does not interfere with 3T3-L1 cell culture. Pen/Strep is not essential for cultivation of 3T3-L1 cells, but can be added without significantly effecting cell growth. Furthermore, we did not observe differences in 3T3-L1 differentiation with and without Pen/Strep. We recommend adding Pen/Strep when the cells are handled by several individuals.

**Table:** Differentiation media 1 (DM1)

Reagent	Stock concentration	Final concentration	Volume
CM	-	-	50 mL
Insulin	2 mg/mL	1 μg/mL	25.0 μL
Dexamethasone	10 mM	0.25 μM	1.25 μL
IBMX	500 mM	500 μM	50.0 μL
Rosiglitazone	20 mM	2 μM	5.0 μL
**Total**	**N/A**	**N/A**	**∼50.1** **mL**

**Note:** DM1 must sterile filtered (0.2 μm filter) before usage and can be stored up to two weeks.

IBMX might precipitate in cold media, therefore, it is recommended to prewarm DMEM prior to adding it. Rosiglitazone is not mandatory for differentiation of 3T3-L1 cells into adipocytes, however, it was shown that esp. cells of higher passage have a significant better lipid accumulation when rosiglitazone was added.

**Table:** Differentiation media 2 (DM2)

Reagent	Stock concentration	Final concentration	Volume
CM			50 mL
Insulin	2 mg/mL	1 μg/mL	25.0 μL
**Total**	**N/A**	**N/A**	**∼50.0 mL**

**Note**: DM2 must sterile filtered (0.2 μm filter) before usage and can be stored up to two weeks.

**Table:** Oil Red O (ORO) stock

Reagent	Amount
ORO	0.7 *g*
Isopropanol	200 mL

**Note**: Stir over night, sterile filter (0.2 μm filter). Stored at 4 °C the ORO stock can be used up to six months.

**Table:** ORO working solution

Reagent	Amount
ORO stock	60% (v/v)
H_2_O	40% (v/v)

**Note**: Prepare fresh. Incubate for 20 min at room temperature (RT) and filter (0.2 μm filter).

## Step-by-step method details

### Differentiation of 3T3-L1

**Timing:** ∼**14 days**

The (pre)adipocyte model cell line 3T3-L1 has a fibroblast origin.^10^^,^^11^ During adipogenesis, 3T3-L1 cells are differentiating from preadipocytes to mature adipocytes.^1^^,^^12^^,^^13^^,^^14^^,^^15^ Here, diverse cellular processes characteristic for adipocytes such as accumulation of lipid droplets, adiponectin production, and insulin stimulated glucose uptake are induced. For differentiation of 3T3-L1 cells, we recommend to use 3T3-L1 cells in a passage number below 25. All of the following procedures need to be performed under sterile conditions using a safety cabinet. Media and buffers used for cell culture and differentiation should be prewarmed at 37°C.

1. Day 0 of cell culture/day -4 of differentiation.
  ***Optional:*** Day -4 of differentiation is flexible from day -3 to day -5.
  a. Wash, detach, and count the cells like described in before you begin section (cell culture of 3T3-L1 cells).
  b. Seed the cells into a well format of interest. Necessary cell numbers for the different well formats as well as the required amount of media are given in the following table:

**Table:** 

Well format	Media/Well	Cell count
6-well plate	2.00 mL	1 × 10^5^ cells
12-well plate	1.00 mL	5 × 10^4^ cells
24-well plate	0.50 mL	3 × 10^4^ cells
48-well plate	0.25 mL	2 × 10^4^ cells
96-well plate	0.10 mL	1 × 10^4^ cells

    ***Note:*** Always prepare a mastermix containing cells and media.
2. Day 2 of cell culture/day -2 of differentiation.
  a. Verify 100% confluency.
  b. Incubate for further 2 days (37°C, 5% CO_2_).

***Optional:*** Renew CM.

**Figure 1:**
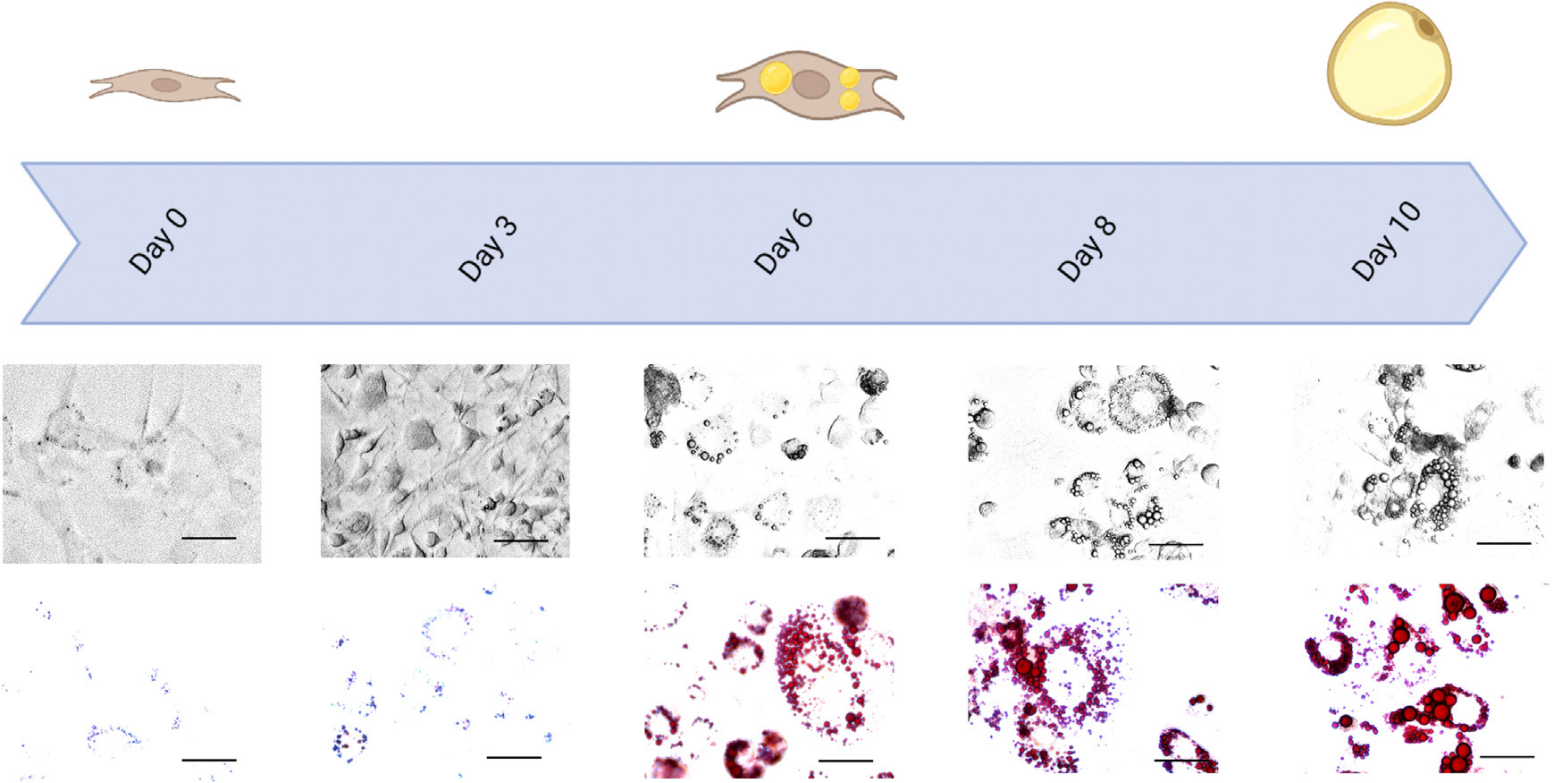
Microscopic pictures of 3T3-L1 (pre)adipocytes during differentiation 3T3-L1 preadipocytes were differentiated into mature adipocytes. Within this process, we took pictures using cells after fixation and ORO stain. On day 0 of differentiation, 3T3-L1 preadipocytes are confluent with a fibroblast like phenotype. Three days later (day 3) maturing preadipocytes are rounder and filaments are getting shorter. On day 6, first lipid droplets are forming and the cells show a round shape. Until day 8, these lipid droplets grow to larger sizes, which is further induced by additional two days of differentiation. On day 10 3T3-L1 adipocytes are fully differentiated with multiple lipid droplets; scale bar: 50 μm.
4. Day 7 of cell culture/day 3 of differentiation (Figure 1).
  a. Change media to differentiation media 2 (DM2).
  b. Incubation for 3 days (37°C, 5% CO_2_).
5. Day 10 of cell culture/day 6 of differentiation (Figure 1).
  a. Change media to CM.
  b. Incubate for 2 days (37°C, 5% CO_2_).

***Note:*** Media will become viscous. Be careful when removing media.

3. Day 4 of cell culture/day 0 of differentiation (Figure 1).
  a. Change media to differentiation media 1 (DM1).
  b. Incubate for 3 days (37°C, 5% CO_2_).
    ***Note:*** 3T3-L1 must have reached 100% confluence two days prior to differentiation start.

6. Day 12 of cell culture/day 8 of differentiation (Figure 1).
  a. Change media to CM.
  b. Incubate for 2 days (37°C, 5% CO_2_).
7. Day 14 of cell culture/day 10 of differentiation (Figure 1). 3T3-L1 cells are fully differentiated into mature adipocytes.

### Qualitative and quantitative analysis of lipid accumulation

**Timing:** ∼**2 h**

The differentiation of 3T3-L1 preadipocytes to mature adipocytes^1^^,^^12^^,^^13^^,^^14^^,^^15^ is influenced by various factors. To analyze the effect of a treatment on adipogenesis, lipid accumulation in the cells can be determined. Thereto, ORO, a lipophilic agent, can be used to stain lipid droplets in (mature) 3T3-L1 adipocytes.^1^^,^^13^^,^^14^^,^^16^^,^^17^

***Note:*** The following steps are described for culture plates in a 48-well format. We recommend seeding three technical replicates per treatment.

8. Fix mature 3T3-L1 adipocytes.
  a. Discard media.
  b. Add 250 μL formalin (10% (v/v) in PBS).
  c. Incubate for 5 min at RT.
  d. Renew formalin.
  e. Incubate for 1 h at RT.

***Optional:*** Substitute formalin with water and wrap plate with parafilm for storage up to 4 weeks (4°C, in the dark).

**Figure 2:**
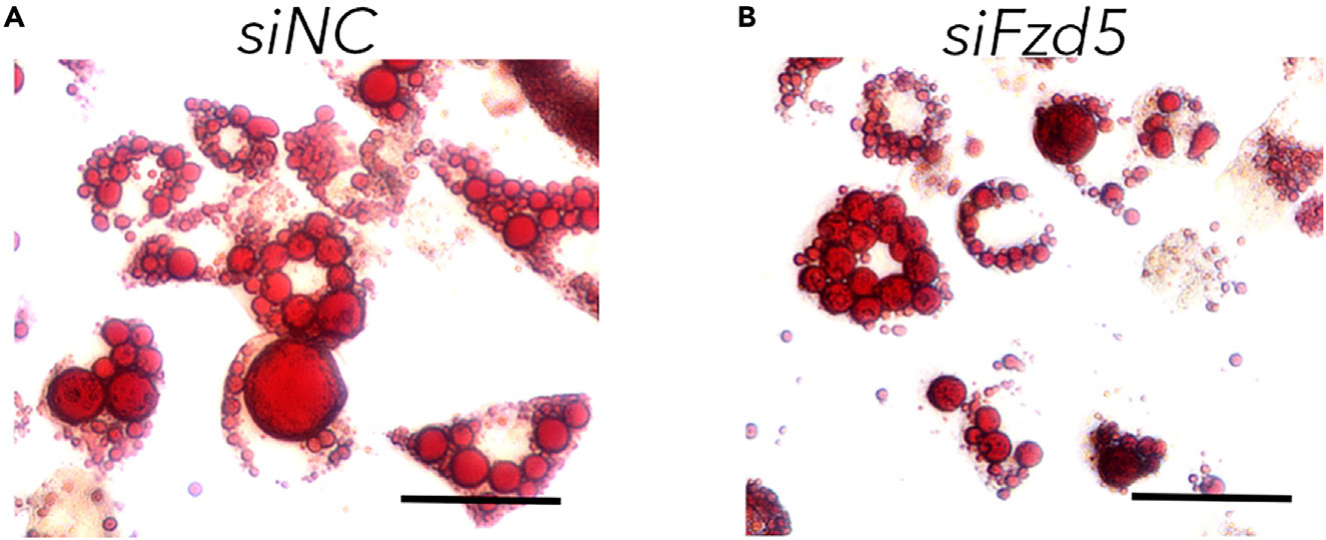
Microscopic pictures of 3T3-L1 adipocytes 3T3-L1 preadipocytes were differentiated into mature adipocytes before lipid droplets were stained using ORO. (A) We previously showed that control transfected adipocytes (*siNC*^1^) are comparable to wild-type cells.^14^ Here, we find around 60% of the cells to be differentiated into mature adipocytes. Many lipid droplets of different sizes, which surround the nucleus, are characteristic for the phenotype of mature 3T3-L1 adipocytes. (B) When reducing expression of *Fzd5* via siRNA-mediated knockdown (*siFzd5*), the pictures show a reduced number and size of lipid droplets already implying a role of *Fzd5* in adipogenesis^1^; scale bar: 50 μm.

**Figure 3:**
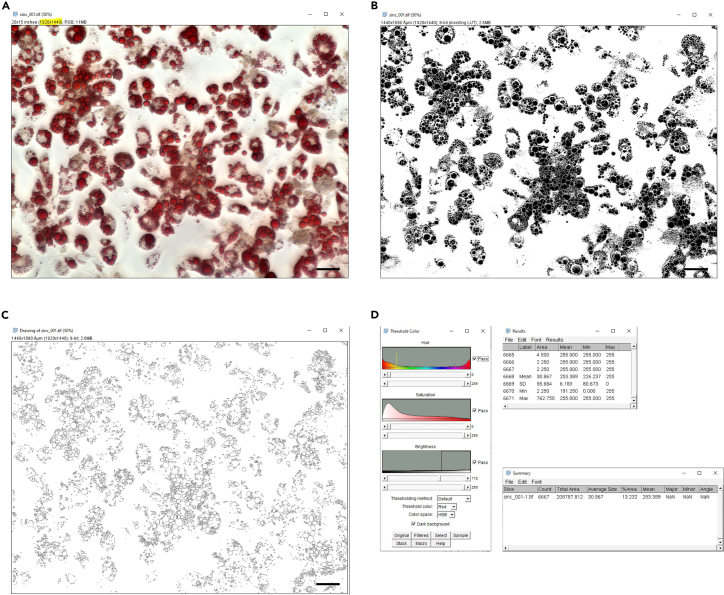
Analyzing microscopic pictures of 3T3-L1 adipocytes in ImageJ 3T3-L1 preadipocytes were differentiated until day 10 of differentiation into mature adipocytes. The following pictures describe the steps within the ImageJ macro in detail. (A) A microscopic picture of mature 3T3-L1 adipocytes was taken. The size of the picture is marked in yellow. (B) Applying the ImageJ macro, exemplarily shown for the picture depicted in Figure 3A, leads to a binarization of the image and separates the partially overlapping droplets by watershed object separation. (C) In the next step, the macro creates a drawing for manual, optical control of the measured droplets. (D) The applied color threshold for the current analysis, the resulting droplet sizes of each individual, recognized droplets, and the summary about all lipid droplets analyzed in the picture (here for Figure 3A) are shown.

9. Stain adipocytes.^1^^,^^10^^,^^13^^,^^14^^,^^16^^,^^17^
  a. Discard formalin.
  b. Wash cells with 250 μL 60% (v/v) isopropanol.
    ***Note:*** Plate can be tapped on paper towels to remove excess liquid.
  c. Let cells dry.
  d. Add 150 μL ORO working solution per well onto the cells.
  e. Incubate for 10 min at RT.
  f. Discard ORO working solution.
  g. Wash cells five times under running distilled water.
    ***Note:*** Do not allow water to flow directly onto the cells to prohibit cell detachment. Hold the plate at a slight angle.
  h. Cover cells with 250 μL distilled water.
10. Take pictures using bright-field microscopy (Figures 2 and 3A).

**CRITICAL:** Do not focus the cells completely. Make sure that the lipid droplets appear round (Figures 2 and 3A). Take pictures with high contrast. Take three pictures per well resulting in nine pictures per treatment (three pictures per well with three technical replicates) to achieve good comparability between different treatments. Do not choose a specific spot but take pictures randomly.

11. Analyze lipid droplet size and number using ImageJ.
  a. Save the following code in ImageJ as macro (derived from Deutsch et al.^8^).
    ***Note:*** Adjust the scale to your microscopic pictures (line 1, set in bold). These values can be found in the upper left corner after opening a microscopic picture in ImageJ (Figure 3A).^1^^,^^13^^,^^14^
    Figure 1: lipid droplets were stained using Oil Red O.
    run("Set Scale...", "distance=**1920** known=**1440** unit=μm");
    run("Color Threshold...");
    min=newArray(3);
    max=newArray(3);
    filter=newArray(3);
    a=getTitle();
    run("HSB Stack");
    run("Convert Stack to Images");
    selectWindow("Hue");
    rename("0");
    selectWindow("Saturation");
    rename("1");
    selectWindow("Brightness");
    rename("2");
    min[0]=0;
    max[0]=255;
    filter[0]="pass";
    min[1]=**132**;
    max[1]=**255**;
    filter[1]="pass";
    min[2]=**70**;
    max[2]=**255**;
    filter[2]="pass";
    for (i=0;i<3;i++){
    selectWindow(""+i);
    setThreshold(min[i], max[i]);
    run("Convert to Mask");
    if (filter[i]=="stop") run("Invert");
    }
    imageCalculator("AND create", "0","1");
    imageCalculator("AND create", "Result of 0","2");
    for (i=0;i<3;i++){
    selectWindow(""+i);
    close();
    }
    selectWindow("Result of 0");
    close();
    selectWindow("Result of Result of 0");
    rename(a);
    setOption("BlackBackground", false);
    run("Make Binary");
    run("Watershed");
    run("Analyze Particles...", "size=2-Infinity circularity=0.50-1.00 show=Ellipses display exclude clear include summarize");
    run("Summarize");
  b. Install macro (Plugins→Macro→Install).
  c. Open one of the microscopic pictures in ImageJ.
  d. Run macro (Plugins→Macro→Run).
    ***Note:*** Exemplary pictures of the results are depicted in Figures 3B‒3F. Check if lipid droplets were recognized thoroughly (Compare Figures 3A and 3C). If not, adjust saturation (filter 1, lines 20 and 21, set in italics) and/or brightness (filter 2, lines 23 and 24, underlined) of the macro by changing the min and max values.
  e. Repeat steps 11.c and 11.d for all other pictures.
  f. Use the results gained from the macro (Figure 3D) to analyze lipid droplet number and size (Figure 4).^1^^,^^13^^,^^14^

**Figure 4:**
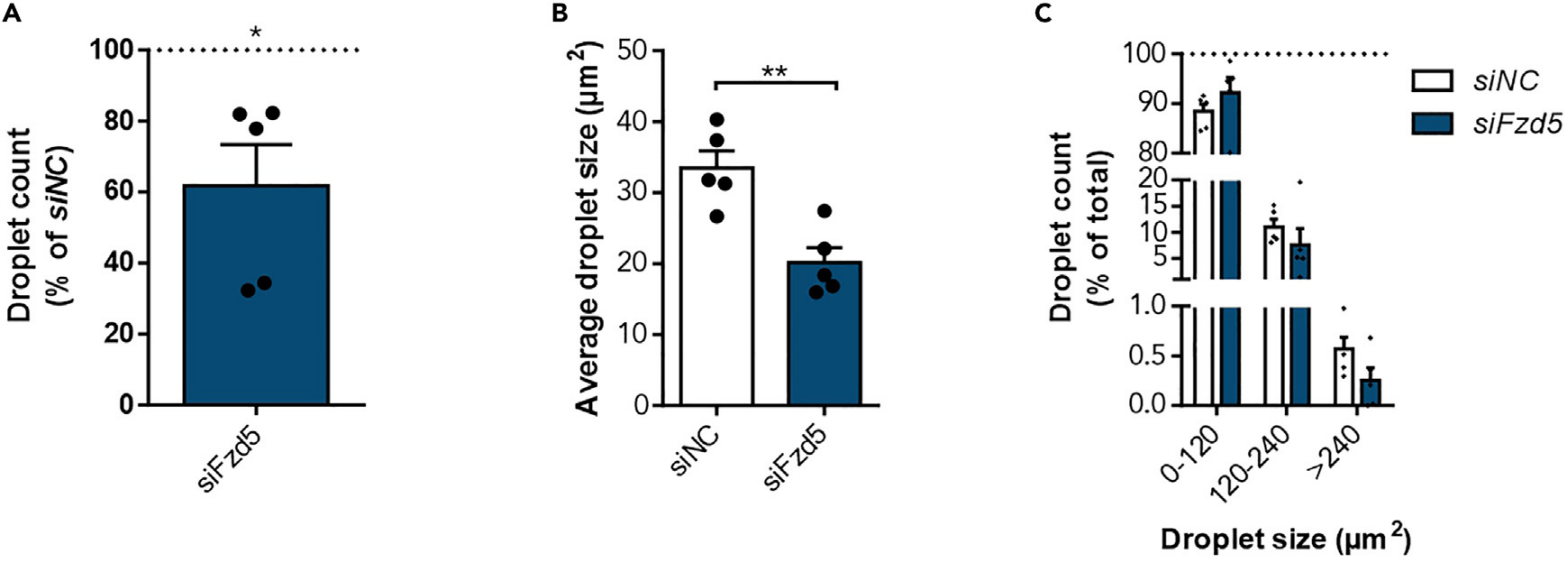
Lipid droplet analysis of the microscopic pictures in ImageJ After differentiating 3T3-L1 preadipocytes into mature adipocytes and ORO staining, microscopic pictures were taken and analyzed using ImageJ and the respective macro. (A) The obtained data were used to calculate the droplet count per picture. To identify, if *siFzd5* influenced adipogenesis, we calculated the relative droplet count compared to *siNC* (n_lipid droplets_ = 6910.8 ± 925.9) and found *siFzd5* to reduce the amount of lipid droplets significantly. (B) Analyzing the average droplet size per picture, we found *siFzd5* to reduce the droplet size. (C) For further clarification of the impact of *siFzd5* on adipogenesis, lipid droplet size distribution was calculated. Here, we found a non-significant increase of small lipid droplets (0–120 μm^2^), as well as a trend to less middle-sized and big lipid droplets (120 μm^2^–240 μm^2^, >240 μm^2^). Summarized, lipid droplet analysis using ORO provides information about the lipid droplet number, average size, and size distribution. Shown is the mean ± SEM of five independent experiments. Statistical significance was identified using a paired Student’s t test (A and B) or a Two-way ANOVA (C).^1^
12. Elute ORO stain from the cells for quantification of lipid accumulation.
  a. Discard water.
  b. Let cells dry.
  c. Add 150 μL 100% (v/v) isopropanol per well to elute ORO.
  d. Incubate for 10 min at RT.
  e. Pipette solution up and down.
  f. Transfer 140 μL of eluted ORO into a fresh 96-well plate (flat bottom).
  g. Measure eluted ORO at OD_500 nm_ and correct for OD_620 nm_ (Figure 5).^1^^,^^13^^,^^14^^,^^16^

**Figure 5:**
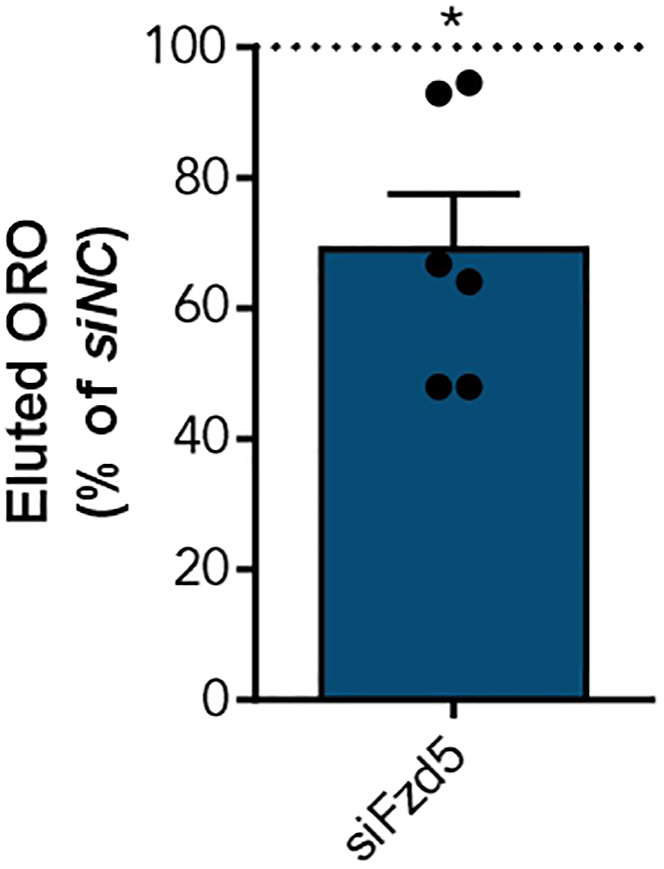
Evaluation of total lipids accumulated into the cells 3T3-L1 preadipocytes were differentiated into mature adipocytes before ORO staining. Afterwards, ORO was eluted to calculate the accumulation of lipids into the cells relative to control (OD_500nm_ = 0.40 ± 0.03). Shown is the mean ± SEM of six independent experiments. Statistical significance was identified using a paired Student’s t test.^1^

## Expected outcomes

Over the time course of differentiation, 3T3-L1 cells change their morphology and incorporate lipid droplets (Figure 1).

Different treatment methods can have an impact onto lipid accumulation leading to increased or reduced formation of lipid droplets. After ORO staining these changes are visible in bright-field microscopic pictures. In Figure 2, application of a specific siRNA demonstrates the effect a gene has onto 3T3-L1 adipogenesis.^1^

Changes in the amount of incorporated ORO due to reduced lipid accumulation can be caused by formation of a lower number and/or a reduced size of droplets. These changes can be evaluated using the ImageJ macro. The initial bright-field microscopic picture (Figure 3A) is converted by color thresholding, binarization, inversion, and watershed object separation (Figures 3B and 3C) before calculating droplet size (Figure 3D).

Those obtained values can be used to calculate changes due to the applied treatment method (Figure 4). This will show if observed changes in lipid accumulation are a result of a reduced droplet number (Figure 4A), a reduced droplet size (Figure 4B), or a combination of both. In-depth analysis of droplet size distribution can also be performed (Figure 4C). These data will help to interpret observed changes in adipogenesis, which is quantified by measuring eluted ORO (Figure 5).

## Quantification and statistical analysis

1. Analysis of lipid droplet number (Figure 4A).
  a. Take droplet count for every treatment group.
    ***Optional:*** Calculate the percentage compared to control treated/differentiated cells.
  b. Statistical significance can be determined using a paired Student’s t test to account for batch-effects in differentiation.
2. Investigating the average lipid droplet size (Figure 4B).
  a. Take the size of each lipid droplet for every treatment group.
  b. Calculate the mean droplet size for each of the different treatment groups.
  c. Statistical significance can be determined using a paired Student’s t test to account for batch-effects in differentiation.
3. Analysis of lipid droplet size distribution (Figure 4C).
  a. Take the size of each lipid droplet for every treatment group.
  b. Sort the lipid droplets into different size groups (e.g., 0–120 μm^2^, 120–240 μm^2^ and <240 μm^2^).
  c. Calculate the percentage of the droplets per size group by dividing the respective droplet count by the total droplet count.
  d. Statistical significance can be determined using Two-way ANOVA when performing multiple comparisons.
4. Quantification of ORO staining (Figure 5).
  a. To account for batch-associated differences in 3T3-L1 adipogenesis, it is also an option to calculate the percentage of eluted ORO compared to control treated cells.
  b. Statistical differences between two treatments can be assessed using a paired Student’s t test to compare the eluted amount of ORO.

## Limitations

3T3-L1 differentiation from preadipocytes to mature adipocytes has multiple advantages. Since it is a preadipocyte model cell line with adipogenic potential, adipogenesis as well as (pre)adipocyte functionalities can be analyzed *in vitro*. Also, lipid accumulation into the cell can be determined using ORO. However, these methods show three limitations. First, 3T3-L1 cells are a model cell line derived from mice, which is cultured in 2D and as a monoculture. As adipose tissue is three-dimensional and consists of diverse cell types (e.g., adipocytes, immune cells, endothelial cells), 3T3-L1 can only depict the functionality of (pre)adipocytes partly. Second, analyzing lipid droplet count per picture, we cannot identify the droplet number per cell. Third, lipid droplets smaller than 2 μm^2^ cannot be detected using the ImageJ macro.

## Troubleshooting

### Problem 1

Differentiation media DM1 contains precipitates (differentiation of 3T3-L1, Step 3).

### Potential solution

• Prewarm DM1 at 37°C to solubilize potential IBMX precipitates.

OR

• Prepare fresh DM1. To ensure IBMX solubility, prewarm DMEM prior to adding this substance.

### Problem 2

3T3-L1 preadipocytes do not differentiate well into mature adipocytes (differentiation of 3T3-L1, Step 7).

### Potential solution

• If you stored differentiation media for more than two weeks, prepare fresh media and repeat differentiation.

OR

• If the passage of 3T3-L1 is high (above 25), thaw fresh cells and repeat differentiation.

OR

• If cells in culturing flask became too dense and lost the adipogenic potential, thaw fresh cells and repeat differentiation.

OR

• If differentiation is started earlier than two days post cell confluence, seed new cells, check cell confluence and induce differentiation two days after reaching 100% confluence.

### Problem 3

After ORO staining precipitated ORO can be seen microscopically (qualitative and quantitative analysis of lipid accumulation, Step 10).

### Potential solution

• Prepare fresh ORO working solution, filter properly, and use on newly differentiated cells (qualitative and quantitative analysis of lipid accumulation, Step 9d).

### Problem 4

After ORO staining, lipid droplets cannot be detected microscopically (qualitative and quantitative analysis of lipid accumulation, Step 10).

### Potential solution

• Cells are not differentiated well. Please refer to problem 1.

OR

• ORO stock and working solution are not prepared according to the protocol (materials and equipment).

### Problem 5

Using the Image J macro, lipid droplets cannot be analyzed properly (qualitative and quantitative analysis of lipid accumulation, Step 12).

### Potential solution

• Adjust the macro like described in Step 11d (qualitative and quantitative analysis of lipid accumulation).

OR

• Take microscopic pictures with a higher contrast and exposure time (qualitative and quantitative analysis of lipid accumulation, Step 10).

### Problem 6

Microscopic pictures and eluted ORO do not fit together (qualitative and quantitative analysis of lipid accumulation, Steps 10, 11 and 12).

### Potential solution

• Microscopic pictures were not taken randomly and, therefore, do not represent the differentiation status within the well.

OR

• Measurement of optical density was carried out with an incorrect wavelength.

OR

• Eluted ORO was not transferred into a fresh 96-well plate (qualitative and quantitative analysis of lipid accumulation, Step 12f).

## Resource availability

### Lead contact

Further information and requests for resources and reagents should be directed to and will be fulfilled by the lead contact, Doreen Thor (doreen.thor@medizin.uni-leipzig.de).

### Technical contact

Questions about the technical specifics of performing the protocol should be directed to and will be answered by the technical contact, Isabell Kaczmarek (isabell.kaczmarek@medizin.uni-leipzig.de).

### Materials availability

This study did not generate new unique reagents.

### Data and code availability

This study did not generate datasets. The generated code is depicted in the manuscript.
